# When incentives work too well: locally implemented pay for performance (P4P) and adverse sanctions towards home birth in Tanzania - a qualitative study

**DOI:** 10.1186/1472-6963-14-23

**Published:** 2014-01-18

**Authors:** Victor Chimhutu, Ida Lindkvist, Siri Lange

**Affiliations:** 1 Department of Health Promotion and Development (HEMIL), University of Bergen, P.O. Box 7807, Bergen 5020, Norway; 2 Chr. Michelsen Institute (CMI), P.O. Box 6033, Bergen, Bedriftssenteret 5892, Norway; 3 Lindkvist is currently employed at Norad, P.O. Box 8034 Dep, Oslo 0030, Norway

**Keywords:** Payment for performance, Results-based financing, Motivation, Tanzania, Mvomero, Home birth, Working conditions, Public health, Reproductive health, Maternal health

## Abstract

**Background:**

Despite limited evidence of its effectiveness, performance-based payments (P4P) are seen by leading policymakers as a potential solution to the slow progress in reaching Millennium Development Goal 5: improved maternal health. This paper offers insights into two of the aspects that are lacking in the current literature on P4P, namely what strategies health workers employ to reach set targets, and how the intervention plays out when implemented by local government as part of a national programme that does not receive donor funding.

**Methods:**

A total of 28 in-depth interviews (IDIs) with 25 individuals were conducted in Mvomero district over a period of 15 months in 2010 and 2011, both before and after P4P payments. Seven facilities, including six dispensaries and one health centre, were covered. Informants included 17 nurses, three clinical officers, two medical attendants, one lab technician and two district health administrators.

**Results:**

Health workers reported a number of strategies to increase the number of deliveries at their facility, including health education and cooperation with traditional health providers. The staff at all facilities also reported that they had told the women that they would be sanctioned if they gave birth at home, such as being fined or denied clinical cards and/or vaccinations for their babies. There is a great uncertainty in relation to the potential health impacts of the behavioural changes that have come with P4P, as the reported strategies may increase the numbers, but not necessarily the quality. Contrary to the design of the P4P programme, payments were not based on performance. We argue that this was due in part to a lack of resources within the District Administration, and in part as a result of egalitarian fairness principles.

**Conclusions:**

Our results suggest that particular attention should be paid to adverse effects when using external rewards for improved health outcomes, and secondly, that P4P may take on a different form when implemented by local implementers without the assistance of professional P4P specialists.

## Background

Performance-based payments (P4P) are seen by leading policymakers as a potential solution to the slow progress in achieving the main target of Millennium Development Goal 5: reducing the maternal mortality ratio by two-thirds by 2015 [1-3]. The basic principle behind P4P is that payments are contingent on performance. For example, an increase in the utilization of health services can trigger a bonus to the responsible health workers/managers [4]. It is assumed that payments to health-care providers will induce them to offer maternal health-care services of a higher quality, and that this in turn will increase the number of women and children who receive high-quality care. In Africa, 17 countries are now implementing P4P, 14 of them as pilots, and three as nationwide programmes [5].

Critics argue that P4P need not result in improved health outcomes, and that it can have adverse effects [6-10]. In particular, P4P may crowd out motivation and/or attention for tasks for which health workers are not paid. Health workers may also play with the books and alter numbers and not behaviour. Lastly, P4P programmes are criticized for focusing on the quantity- rather than the quality of care.

Proponents of P4P refer to a relatively limited number of studies that demonstrate that P4P can have a substantial effect on the utilization of health-care services and to some extent on health outcomes [11-15]. A weakness of these studies, however, is that they do not tell us precisely *how* or *why* these changes take place. If we obtain a better understanding of what health providers think about P4P programmes [16], and what they actually do to make changes occur, it will help us shed some light on any potential adverse effects, as well as helping us understand why P4P appears to work well in some settings and not in others.

The majority of P4P studies that have found positive results in terms of utilization and/or outcomes have used data from programmes in which substantial technical and managerial resources accompany the intervention. In countries with weak health sector institutions, this is unlikely to be the case during nationwide roll-out organized by both national and local governments. It is therefore important to also study interventions that are implemented by governments in low-income countries using their own resources.

This paper offers insights into two of the aspects lacking in the current literature on P4P: Why changes may occur in response to the introduction of P4P, and how the intervention plays out when implemented by local government as part of a national programme that does not receive donor funding. The paper is structured as follows: The first section looks at the underlying conceptual framework of P4P, while the second section describes the nationally funded P4P programme in Tanzania. The third section lays out the methods that we employed, whereas the fourth section reports on the findings from the in-depth interviews with health workers and health administrators. The fifth and sixth sections offer a discussion of the results and concluding remarks.

### Conceptual framework

Payment for performance (P4P), or performance-based payment (PBP), can be defined as: “the transfer of money or material goods conditional upon taking a measurable action or achieving a predetermined performance target” [17]. Underlying P4P is the *principal*-*agent* model. The principle behind this model is that there is a lack of alignment of the preferences (interests) of the *principal* (employer) and the *agent* (employee) when it comes to the goals to be achieved by an organization. The *principal* therefore attempts to find ways of aligning the *agent’s* goals to the goals of the organization [18]. In the context of P4P, health workers, or *'agents’ ,* are provided with performance bonuses by the *principal* in order to achieve health outputs and outcomes.

Nevertheless, even if a health worker is strongly motivated by the reward the principal offers and changes his behaviour in response, the intervention may not necessarily improve health outcomes.

First, financial incentives may crowd out attention to tasks important for high-quality care. Health workers may focus on aspects of health care for which they are rewarded, while ignoring other aspects of care for which they are not rewarded yet are nonetheless important for quality [7]. Holmstrom and Milgrom coin the former type of behaviour multitasking, and argue that financial incentives may not be very effective in the health sector, as employers pay for input rather than for health outcomes [19].

Second, external rewards may crowd out health workers’ intrinsic motivation to do the job [20]. Clearly, if health workers were intrinsically motivated to deliver high-quality health care, there would be no need for employing financial incentives as a motivator. Studies comparing what health workers in low-income countries *can* do with what they actually do [21-23] suggest that the intrinsic motivation for the average health worker is low. However, the use of external rewards may still crowd out the motivation for those health workers who *are* intrinsically motivated, and if the use of external rewards for some reason is discontinued, motivation and effort may end up being lower than before these rewards were introduced.

Third, P4P is vulnerable to corruption, i.e. health workers may be changing the numbers rather than the indicators themselves (see Oxman and Fretheim for a review of adverse effects [6]. Fourth, a major assumption of P4P is that workers are able to offer high-quality care if they choose to. This may not be the case, as knowledge of guidelines and access to equipment and medication may be inadequate. Lastly, P4P presupposes that if health workers deliver high-quality care, women will come to the facility. This may not be the case since women may deliver at home for reasons outside the control of health workers.

In conclusion then, agency theory tells us that offering health workers an incentive for an increase in the number of deliveries need not result in improved health outcomes, primarily because the incentive is related to effort and not to outcomes.

### P4P in a Tanzanian context

The productivity of health workers in Tanzania has been proven to be low. One study shows that less than 60% of working hours are used for productive activities, ([24]:3) whereas another demonstrates that few health workers follow clinical guidelines, and that low motivation is a central factor [23]. Lastly, a number of studies have shown that health workers in Tanzania are unhappy with their working environment and their salaries [25,26].

Norway, one of Tanzania’s long-term development partners, took a leading role in introducing the idea of result-based financing in the country’s health sector in 2008 [2,27]. The government of Tanzania was very receptive to the idea and wanted to launch a national P4P pilot programme in 2009 [28]. Tanzania’s development partners in the health sector were reluctant to endorse the idea due to many contested issues. First, there was a strong feeling that the state of Tanzania’s health management information system (HMIS) was not ready for P4P, which had been documented in an appraisal study carried out in 2009 [2]. Second, other perceived preconditions for a successful P4P, such as a satisfactory staff situation and adequate access to essential drugs, equipment and supplies, were lacking [2]. The government of Tanzania was therefore not allowed to use the funds in the health basket earmarked for P4P. While acknowledging that the proper conditions for P4P were lacking, the government of Tanzania proceeded with the implementation of P4P in 2009, choosing to employ a 'learning by doing approach’ [28].

The donor community requested that the government of Tanzania halt P4P [27]. At this point, however, the government of Tanzania had already issued a directive that P4P should be included as an activity in the districts’ Comprehensive Council Health Plan (CCHP) for 2009/10. Even so, not all the districts followed the directive, and in some places health workers were eagerly awaiting a P4P programme that was never implemented [29]. On the other hand, the district administration in Mvomero District decided to follow the directive and P4P was consequently budgeted for in the health plan and implemented in 2009, and health workers received their first bonuses in 2010. To distinguish the P4P scheme that we study from the donor-funded scheme which was later launched as a pilot in Pwani Region in 2011 [30], we will refer to it as the 'locally funded P4P’.

### The design of the locally funded P4P in Tanzania (2009–2011)

The main aim of the locally funded P4P in Tanzania was to “provide better motivation and explicit attention to results, by ensuring that health workers and their supervisors are motivated to strive for better results in Maternal, Newborn and Child Health Services and other health services in the districts” [28]. The bonuses were to be paid based on achievements using the following indicators: antenatal care, institutional deliveries, post-natal care, and Health Management Information Systems (HMIS). Council Health Management Teams (CHMTs) were to monitor and ensure that health facilities in their mandated area were submitting their reports in time, and they were also to review and verify the reports. In turn, the CHMTs were to be monitored by the Regional Health Management Teams (RHMTs) [31].

At the facility level, a maximum annual bonus was to be achieved if the facility met all the targets for all the indicators, whereas a partial bonus was to be paid if only some of the targets were met. For deliveries, the target for dispensaries was that 60% or more of all the expected deliveries of the catchment area should take place at the dispensary. At the national level, 51% of all women with a live birth received delivery care from a skilled provider in 2010, although the percentages vary between 21% and 91% across regions [32]. New targets were to be set at the beginning of each year and the basic rule for target setting was the requirement of improvements from the previous performance [31]. Furthermore, the bonuses were to differ according to facility type. Dispensaries had a maximum bonus limit of T.Shs 1 million (approximately USD 676) and health centres, CHMTs and RHMTs’ maximum annual bonus was T.Shs 3 million (USD 2,000), while hospitals had the highest maximum annual bonus of T.Shs 9 million (USD 6,000). Payments at the health facility were to be shared equally among the staff regardless of grade, qualifications or position. If a health facility reached all targets, each individual was supposed to get a maximum annual bonus of approximately T.Shs 200,000 (USD 136) [31].

## Methods

### Study context

The study was conducted in Mvomero, a rural district in the Morogoro Region of Tanzania. The district covers more than 7,000 square kilometres, and the population is approximately 300,000. Administratively, Mvomero is divided into 17 wards and 101 villages. There are 56 health facilities in the district, including three hospitals, four health centres and 49 dispensaries [33]. Six out of 10 live births in the region are delivered by a skilled provider [32]. Except for a woman’s first delivery and her fifth delivery and upwards (which should take place at a hospital), Tanzanian health authorities recommend that women with uncomplicated pregnancies who have gone through regular antenatal care deliver at their closest dispensary. Dispensaries are usually headed by a Clinical Officer (three years of medical training). As a general rule, nurses are in charge of deliveries, but the Clinical Officer will be asked to assist if a delivery does not proceed normally.

### Data collection and analysis

Since we were interested in health workers’ perceptions and experiences with P4P, as well as their strategies to meet set targets, we chose individual interviews as our primary methodology. Moreover, because nurses are in charge of the majority of services targeted by the locally funded P4P (antenatal care, deliveries, postnatal care and vaccinations), we focused on this profession. In June 2010, when P4P had been introduced but payments had not yet been made, the first author conducted 12 in-depth interviews with health workers at four public dispensaries and one faith-based health centre (10 nurses, one medical attendant and one lab assistant). The interview guide was informed by the existing literature on P4P and focused on expectations related to the introduction of P4P, including the potential effect of P4P on the prioritization of work within the health facility, nurses’ perceptions and experiences of midwifery and provision of care, in addition to perceptions about the access to-, acceptability of- and quality of care provided to women in childbirth.

In October 2011, after health workers had received their first bonus payments, the third author conducted a total of 14 IDIs with health workers at five public dispensaries (including a total of three clinical officers, 10 nurses and one medical attendant). Three of the facilities and three of the nurses had been part of the 2010 study. The interview guide covered the following themes: health worker’s knowledge about the P4P scheme (goals, rewarded tasks), actions that had been taken to reach the goals (particularly in relation to increasing deliveries), multitasking, perceptions about the bonus that had been received and how it had been spent, and the perceived effects on cooperation within the facility and communication with the district authorities.

Health workers were specifically asked about sanctions against women who give birth at home. The background for including this question was information that we had gained through focus group discussions (FDGs), which were conducted with the help of research assistants in the period from July to early October 2011. A total of 11 focus group discussions (six with women, five with men) in four different villages were conducted, focusing on the perceptions of maternal health services in the district and the potential benefits and challenges of P4P. Due to the scope of the journal article format, the findings of the FDGs will not be included here. However, the FDGs provided important background information for our IDIs with health workers. For example, we learned from two of the FDGs that health workers had announced that any woman who gave birth at home would be fined. Hence, health workers were asked about fining and other forms of sanctions directed at home births.

The health facilities were located between one and three hours by car from each other, mostly in different wards. The facilities were partially on the basis of acquaintance through previous visits, which improved their rapport with informants, and partially on the basis of convenience. In general, all the health workers who were present at a given facility at the time of the fieldwork were interviewed.

In addition to IDIs with health workers and FDGs with community members, we carried out individual in-depth interviews with two district health administrators, including the District Medical Officer. The interviews focused on the process of implementing the government funded P4P, including lessons learned and the reasons why the scheme was discontinued.

All interviews were conducted in Swahili. The first author speaks Swahili on a high level, while the third author is fluent in the language and has conducted a number of long-term ethnographic fieldworks in Tanzania over a period of 20 years. All IDIs were recorded, transcribed and translated to English. In addition, rapid note taking during interviews was done. The third author checked all transcripts and verified the translations. The study used meaning condensation as the mode of analysis [34], which was assisted by the use of software OpenCode 3.6. The transcripts were subjected to a thorough review and systematic coding. After coding, the content was assigned to categories, and central themes were identified from these categories. During the course of the study, relevant national policy and design documents by the Ministry of Health and Social Welfare were reviewed in a systematic manner, including the Payment for performance strategy [28], the Implementation guidelines for payment for performance [35] and the Results-based bonus design, implementation and budget [31].

### Research ethics

Research clearance was granted in Norway through the Norwegian Social Science Data Services (NSD) and in Tanzania through the Ifakara Institutional Review Board (IHI-IRB), the National Institute for Medical Research (NIMR) and the Commission for Science and Technology (COSTECH), and oral consent was given. We have depersonalized the data by labeling the health facilities by letters and the informants by title.

## Results

### Bonuses were given at a flat rate, yet health workers were satisfied with the payments

The P4P bonuses in Mvomero were paid in February 2011. The District Medical Officer (DMO) and the District Treasurer turned up at each facility and distributed the money in cash. Even though health workers had been told that payments were contingent on performance beforehand, this turned out not to be the case. In contrast to the way that the P4P programme was designed (presented above), the bonus was given at a flat rate and was unrelated to the actual results. Nevertheless, we observed that although payments failed to be contingent on performance, we can still expect the scheme to have worked as performance pay, as long as health workers *thought* payments would be contingent on performance. However, future effects are another matter entirely. The district administration gave the following reason for paying the bonuses at a flat rate:

In 2009–10 we paid all facilities, they all qualified. We saw how many vaccinations they had given, if they had brought the reports timely, etc. We also looked at deliveries. Some people qualified in some respects and not in others, and since this was like a motivation for the employees, we just paid all the facilities the same amount (…). For the case of last year, we were not able to pay P4P because it was not in the budget (a new ambulance was prioritized). If we had continued with P4P we would have been stricter, we would have put more efforts to check whether they had really improved or not. (District Administrator, 2011)

Each dispensary was given T.Shs 500,000, while each health centre was given T.Shs 700,000 to be equally distributed to all staff members at the facility regardless of rank. These bonuses were approximately 50% of the maximum bonuses stated in the P4P planning documents. Depending on the number of staff who shared the bonus at each facility, the actual sums that individual health workers received varied considerably between the facilities we visited, from T.Shs 18,000 to T.Shs 169,000, with the vast majority receiving more than T.Shs 100,000. In comparison, interviewees had a take home monthly salary varying between T.Shs 380,000 to 450,000. The bonus thus constituted 5-40% of what health workers would usually receive per month. Nonetheless, it should be noted that many of our informants had taken loans to finance their children’s education, and after the deductions of these loans the performance bonuses were even more significant. The majority of the health workers used the bonus for their daily requirements, while three had used the money to move one of their children to a school that was either of higher quality and/or closer to where they worked.

Informants expressed gratitude for having received the payments. While many said that it was “like a dream”, something that they had hoped for but not fully trusted that they would receive (due to a prior experience of nonpayment of allowances), others expressed that they had been quite confident that they would get the bonus:

I wasn’t surprised, as we were told that if we do well this would follow. (Nurse 3, Dispensary C, 2011, received 60,000)

Some respondents brought in the question of unpaid overtime, saying that P4P showed that the government actually cares about them after all:

I think P4P is good because it motivates the workers and makes them realize that the government cares about them. (CO, Dispensary C, 2011, received 169,000)

While some informants hinted that the bonus should preferably have been a bit higher, others emphasized that the bonus was a gift (*zawadi*) and not part of their regular salary, and therefore not something that they could demand:

I was so glad to see that our superiors considered us, the people at the bottom. (…) And whatever a father decides to give to his son – that is something that cannot be forced. Personally, therefore, I am so happy to have received that reward and I saw the sum as large and satisfactory because I did not expect it to happen. (Nurse 2, Dispensary B, 2011, received 100,000)

None of the interviewed health workers had been informed about the discontinuation of P4P, and many said that they expected new bonus payments to be made. The district administration confirmed that they had not sent out any written information about the discontinuation of the scheme, but that they had attempted to orally inform health workers during supervisions.

#### Attitudes toward alternative usage of the funds changed after payments were made

In 2010, before the bonus payments had been made, a good number of our informants expressed scepticism towards the idea of P4P. Some informants warned against rewarding health workers in isolation:

P4P is just addressing health workers, but we do not work alone or with no assistance. For example, we are assisted by traditional birth attendants and traditional healers. But if the government had said, OK, we have some incentives for these traditional health workers…ohh surely we would not miss these targets. (Nurse 1, Dispensary D, 2010)

In 2011, a number of our informants said that they had approached Traditional Birth Attendants (TBA) to encourage them to bring women in labour to a health facility, though none argued that TBAs or healers should be given P4P. Another area where the tone has changed is the question of equipment. A number of studies have shown that dispensaries in Tanzania often lack equipment and medicines, and health workers often identify a lack of laboratories as a major hindrance for offering quality care [23]. In 2010, some informants argued that the resources that were to be spent on P4P would have little effect if the equipment situation was not improved as well:

I think P4P will help, but first of all the equipment situation at the facilities has to improve. You know, so that P4P can be successful. But if there is no equipment, we will not be able to give the proper treatment and care needed, and then you can’t reach the target. (Nurse 1, Dispensary C, 2010)

After the bonus payments had been made, however, all but one of our informants were in favour of this way of spending health funds. When asked whether some of the money should instead be used for equipment, the respondent quoted above had this to say:

The fact is that it (P4P) is a good approach. It’s an encouragement since working in a village is tedious. Therefore, when they decided to encourage us ….that was really motivating. It should be continued and it shouldn’t be stopped (in favour of buying equipment). (Nurse 1, Dispensary C, 2011, received T.Shs 38,000)

Other informants were more outspoken when arguing that P4P was a good investment compared to spending money on equipment:

Equipment…? For what? We’re really not in need of equipment. (Nurse 1, Dispensary A, 2011, received T.Shs 125,000)

In 2010, some informants expressed concerns that P4P could lead to unethical behaviour in health care. These health workers felt that P4P was aiming at making them more result-oriented, thereby forcing them to prioritize results over quality care. One nurse expressed this reservation in the following way:

*It is not good to use targets. For example, if you are told to treat 100 patients per day, I don’t think you will treat them accordingly, you will just rush them to reach the right number. I don’t think it’s a good idea to put targets in health care.* (*Lab Assistant, faith based Health Centre, 2010)*

When asked whether the forging of data was another possible pitfall of P4P, several informants agreed that this could be a real danger:

They have to think of something that can motivate us and not the P4P-way which says 'when you produce this, then we will give you a bonus’. I think it creates problems where people will forge data at health facilities to meet the target. (Nurse 1, Dispensary A, 2010)

In 2011, the tone had changed considerably. While the above informant said that there may be a chance of forgery since “people need money and some aren’t trust worthy”, the great majority argued strongly that for practical reasons forging data is impossible. Similarly, while many informants agreed in 2010 that P4P might make health workers concentrate on tasks that are rewarded at the expense of other tasks (crowding out), no informants saw this as a problem in 2011.

### Informants claim that P4P has improved services, enhanced cooperation and fostered a spirit of competition

Many informants view P4P as a competition between health facilities. They feel that in order to attract clients, they need to offer better services than other primary health facilities in the same area. In the words of one clinical officer:

P4P is part of a competition and every person wants to be a winner. (…) We have several health facilities and we have the same design and the same indicators, and most health workers will say 'why not here’? Every health facility will want to score better than the other. Due to that spirit, some changes will happen in health service provision. (CO, Dispensary A, 2010)

In 2011, when asked whether P4P had entailed competition between facilities, several informants argued that P4P had indeed improved services and that their facility now attracted patients from outside of their catchment area:

P4P has made people be more thorough in the work that they do. Patients decide to go where they find the best treatment. For example, we get patients who aren’t happy with the services elsewhere and they come to our facility for better treatment. We get expecting mothers who were supposed to be treated at Mkindo, but we can’t tell them to go back. (Nurse 1, Dispensary A, 2011)

Poor staff attitudes have been noted in the literature as one of the reasons why expectant mothers prefer to deliver at home without assistance from qualified personnel as opposed to facility delivery. In 2010, nurses explained that they sometimes had to “be a bit hard” on women who were delivering or to raise their voice:

*You know patients are so different and difficult. A mother may come in the labour room at the stage of contractions. Some of them get confused, so when you try to tell her something and she doesn’t understand or refuse* […] *then sometimes you have to be a little bit hard on her. This is to avoid infections and to help her deliver the baby safely. (Assistant Nurse 1, Dispensary B, 2010)*

In 2011, nurses still argued that they often needed to be strict with women who come to give birth, that women could not choose birthing position but had to lie down on the delivery bed, and that they would generally not allow relatives to enter the delivery room. At the same time, positive staff attitudes were seen by health workers as one of the strategies to increase service utilization, which would again enable them to meet P4P targets. As one medical attendant puts it:

*If you are being given incentives you need to realize that you must have good attitudes towards work and desist from bad behavior, like using bad language to clients. If you do not change you will let your workmates down.* (*Medical Attendant 1, Dispensary C, 2011, received T.Shs 18,000)*

P4P then, appears to have encouraged unity of purpose at the health facility level since the programme was designed to offer incentives to individuals as a result of team performance.

### Strategies to make women deliver at facilities

An important aim of the second round of interviews was to find out what measures, if any, health workers had taken to make more women deliver at their facilities. Some informants say that they have increased outreach activities and sensitized women on the dangers of giving birth at home:

They (women) come here because we sensitize expecting mothers (…) We tell them that here the equipment is sterilized well compared to that used at home, which is not sterilized. They just hang the gloves to dry which is very risky, it can lead to AIDS transmission. Once they hear that they can get AIDS, they come in large numbers. (Registered Nurse 3, Dispensary C, 2011, received T.Shs 60,000)

One informant said that such health education made some women decide to deliver at a health facility, even if her husband or relatives did not see the importance and wanted her to deliver at home. However, the need to attract more clients in order to reach P4P targets has entailed that many health facilities in the district have developed negative strategies to attract and/or force clients to utilize their services. In fact, the staff at all of the five facilities we visited in 2011 admitted that women had either been told that they would be fined if they delivered at home, or that they would be denied a live birth card and/or vaccination for their newborn.

As for fining, focus group participants in the catchment areas of dispensaries E and F told us that health workers had announced that women who delivered at home would be fined T.Shs 10,000. The health workers we interviewed confirmed that this had been announced, but that it was only a threat, and not something that was actually carried out:

Last year there was a clinical officer who used to tell women that if they give birth at home they will be charged a fine, and that they should come to give birth at the dispensary because it is free of charge. He was just saying that to scare them. (…) If you tell them that, they are afraid to get the fine. So up till this day there is no one who gives birth at home. (Assistant Nurse 2, Dispensary F, 2011)

The reason why the fining had not been carried out was that in order to actually fine someone, one would need to involve the Village Government, which had not yet been done. The health workers got the idea to criminalize home birth from colleagues from a neighbouring district, Morogoro Rural, where fines for home birth had been passed as a by-law by local authorities:

When we go for seminars we sometimes ask what others do to sensitize the people. So whatever you hear from others, if you haven’t tried it yet, then you should also attempt it to see how it works, (…) but only after involving the village leaders. There are some things - even if it hasn’t been decided at the district level – that we can make a decision about and then involve the village leadership. (Registered Nurse 1, Dispensary E, 2011)

Other health workers argued against fining, but were positive towards other forms of sanctions:

I don’t think fining the mother is a good approach. We should rather educate women by telling them the consequences of delivering at home. There are many ways to mobilize them like telling them that they won’t be given a birth card at the dispensary (…). Instead they will have to get it from the Ward Executive Officer, a more tasking process. If she doesn’t do that, she will end up not getting a clinic card for the baby. We can manage to mobilize them to a large extent using these threats. (Nurse 3, Dispensary C, 2011)

Also in this case, the strategy was something the health workers had learned from others:

We heard that at “Dispensary B” they have more deliveries these days, so we asked ourselves how are they doing it? How come they are getting safe deliveries, how are they doing it? We investigated how they succeed to have safe deliveries. How come there are fewer midwives, but they perform better? (…) After inquiring about their successes we are now educating our colleagues. (…) If she won’t deliver here, then she won’t get a clinical card for the baby. So it is just an educational competition. But there is no forcing of the mothers. (Nurse 2, Dispensary C, 2011)

At dispensary A, the same approach had been adopted, but the informant felt that it hadn’t had the expected outcome:

We said that for those who deliver at home - their babies won’t be vaccinated and the babies won’t get clinical cards, but they still continued to deliver at home. Maybe we should look for other means, like fining them. We should tell them that those who deliver at home will be fined. (Nurse 1, Dispensary A, 2011)

Health workers, particularly those with a low level of education, appeared to have little scruples about sanctioning women who deliver at home. The various methods were weighted against each other on the basis of the degree to which they were feasible in practice. A medical attendant had the following response to whether fining would be a good way to make women deliver at a facility or not:

The community here is different from that one (where they fine patients). Here, people are quite tricky - just a minor thing and he/she will go to the village administration or to the Councillor to report! You ask yourself why you should cause all that? (…) But fining is good (…), since to be fined - when even money for food is a problem - they will just decide to do what they are told. (Medical Attendant 1, Dispensary C, 2011)

When we asked the district health authorities what they thought about strategies such as fining, their response revealed that they were ambivalent and undecided, and that they did not appear to do anything about these practices:

I don’t know much about it. It is not according to the government guidelines, we don’t have any regulations on that. But people say it helps (…). On the other hand, if the woman doesn’t have the 10,000 shillings she will not take her child to the clinic (for vaccinations, in fear of the fine). (Official from district health office, 2011)

The fact that the council health management team (CHMT) also benefits from health facilities’ good performance may be one reason why the monitoring and follow-up of such practices is limited.

## Discussion

Our findings suggest that health workers did alter their behaviour in response to the intervention. However, to shed further light on how the intervention worked, two questions warrant further discussion: Why did health workers focus on coercive methods to increase deliveries at facilities? And why didn’t the local authorities pay bonuses according to the set targets as outlined in the directives from the Ministry of Health and Social Welfare?

### Change in health worker behaviour

Although the payments ended up not being performance based, health workers were told that they would be, and they responded to the intervention much as the theory predicts that they would: They increased effort in areas where they would receive payments (with a focus on quantity), while strategies to improve the quality of care (for which they were not rewarded) appear to have been largely ignored. Similar results were also reported in a P4P impact evaluation study on maternal and child health in Rwanda where the highest improvements in indicators were observed for those indicators with the highest expected payment [12].

Health workers reported numerous strategies to attract women to the clinics. One of the strategies they reported was to offer better services, educating women about the benefits of delivering at a clinic and positive attitudes toward women attending the clinic. The use of positive strategies to persuade women to give birth at clinics may certainly increase the number of facility births as some studies in Tanzania have found negative staff attitudes to be among the barriers against facility deliveries [36,37]. However, attracting more women to deliver at facilities does not necessarily ensure that the clinical quality of care is high. Due to the difficult circumstances under which many health workers work - lack of equipment, medication, and skills [38] - it may be difficult to deliver high-quality care even if they want to. In fact, prior to the intervention, health workers were afraid that they would not be able to deliver a high quality of care due to a lack of equipment.

Health workers not only engaged in positive strategies to induce women, coercive strategies were also employed, and these strategies were copied between clinics. While other studies have noted that incentive structures may entail coercive strategies, [39,40], to our knowledge, our study is the first to identify coercive practices against home birth in connection with a P4P scheme in Tanzania. Health workers threatened to fine women who did not give birth at a facility or to withhold vaccinations and/or clinical cards for babies who had been born at home. While health workers in Mvomero claim that the threats were meant to “scare” people and that they have not been carried out in actual practice, the district health administration argue that these threats may have kept women from coming to the clinic for postnatal care and vaccinations of their babies after having delivered at home, because they were afraid of being fined.

The effectiveness of both positive and coercive strategies on health outcomes crucially depends on the quality of care offered, which was not directly targeted by the intervention. During our fieldwork in 2011, a woman died of untreated eclampsia because the medical attendant at the dispensary where the woman came to give birth did not recognize the signs of preeclampsia. Since poor quality may be an explanation for why women choose not to deliver at the clinic, health workers could potentially improve the quality to attract more women. However, if health workers offer a low quality care because they are *unable* to deliver a high quality, coercive strategies forcing women to deliver at the clinic may be perceived as the only strategy they have at hand. Such strategies need not improve health outcomes.

Observe that even if health workers reported that they had implemented various strategies to attract women to the clinics, the strategy that would involve the least amount of effort would be to forge the numbers. Before health workers received payments, they were worried that a convenient strategy to fulfill the delivery indicator would be to forge the numbers. After having received the P4P payments, however, health workers were adamant that this did not take place. Certainly, health workers had a vested interest in making this argument. An assessment of the donor-funded pilot undertaken in the Pwani Region suggests that verification and data validation has been a problem and that forging has indeed taken place [41].

### Why wasn’t P4P implemented in the way that it was designed?

Contrary to the directives from the Ministry of Health, the district administration ended up paying bonuses to all facilities at a flat rate, which means of course that the payments were not performance based. The district administration’s explanation for giving a flat rate was that all health facilities did well in at least one indicator, and that they would be “stricter” if P4P was to be continued. In our view, two factors were behind this decision.

First, as documented in an appraisal study commissioned by donors in 2009, the Health Management Information System (HMIS) in Tanzania is too weak to handle a proper monitoring of a P4P programme [2]. A weak health infrastructure has been identified as one of the major threats to the effectiveness of P4P [40]. We witnessed that the health administrators in Mvomero were too busy with other work to take on the additional burden of monitoring P4P. Hence, for an under-resourced district such as Mvomero, monitoring P4P with the help of the district’s own resources probably proved impossible.

Second, the district administration argued that a flat rate was given since the P4P initiative was to be a financial motivation (*motisha*) for the health workers, and that all had done well with at least one indicator. Under P4P, health workers are rewarded for a change in indicators. The extent of change a single facility is able to achieve will be a function of factors both inside and outside health worker control. For a variety of reasons, some facilities will find it difficult to increase the utilization of services (i.e. long distances/lack of transport, strong preferences for home birth among the local population, etc.), while others may find it easier. In clinics where the know-do gap is high and low utilization is explained in part by a low motivation, there may be a high potential for achieving an increase in indicators.

The extent to which bonus payments are perceived as fair – even in cases where they depend upon factors that are partly outside the control of the health workers - depends upon the type of fairness principle that prevails in a particular culture. Based on different ideas about what individuals should be held responsible for, notions of fairness can be divided into two broad categories: libertarianism and egalitarianism [42]. According to libertarianism, performance-based incentives will be viewed as fair since individuals receive payments based on how they perform in relation to a predefined target. On the other hand, according to strict egalitarianism, P4P will be perceived as unfair since people should not be held responsible for factors such as talent or other external aspects outside of their own control. An egalitarian fairness principle may stand strong in socialist societies, where individual behaviour is believed to be shaped by society, and inequality as such is a function of an unfair societal structure rather than being due to any fault of the individual. By contrast, in capitalist societies where ideas of liberalism and libertarianism stand stronger, pay for performance may be viewed as a fair way to differentiate income, which according to libertarianism should be distributed in relation to effort and talent (see Nozick [43] for a defence).

Due to its sociocultural setup and political history, Tanzania is characterized by an egalitarian mode of thought. The local government in Mvomero did not appear compelled to reward good performance and withhold money if the performance was not optimal; instead, they stated that they wanted to display gratitude for what they saw as a positive effort. In addition to the practical limitations mentioned earlier, it is possible that the social relationships between local policymakers and health workers made it difficult for the health administration to pay some facilities less than others even if they had not met the set targets.

Because principles of fairness vary across cultures and countries, P4P may be viewed as fair in some places, while being perceived as deeply unfair in other settings. The literature reports a number of cases where health workers are demotivated by P4P programmes because the rewards are perceived as being unfairly allocated [40]. When we told our informants that the bonuses had been given at a flat rate that disregarded actual performance, none of them argued that this was unfair; nor did informants contest the practice of distributing the bonus equally to all staff members at the facility. One reason for this may be that we focused on nurses. There is the possibility that medical doctors at the three hospitals in the district would have expressed more negative attitudes toward a system where they receive the same bonuses as non-clinical staff.

If it is correct that an egalitarian fairness principle shaped the way that the locally funded P4P was implemented, then P4P may work very differently when implemented by local agents compared to when it is implemented by external entities. The donor-sponsored P4P in the Pwani Region is being piloted and led by professional external agents. Based on our experience from the locally funded P4P in Mvomero, we expect that the intervention may work very differently if/when it is taken over by the government in its entirety.

Our study has a number of implications for the design of P4P in Tanzania and other low-income contexts, and for the studies of such schemes. Firstly, contextual factors affect the nature and operation of a P4P scheme (such as a country’s sociocultural context) and it is therefore important for these factors to be taken into account during the design to achieve sustained results. Secondly, in cases where a P4P design is supply-side oriented (such as the Tanzanian case), there is need to accommodate and promote community views and participation in order to safeguard against coercive practices. Thirdly, our study to a certain extent implies that P4P is prone to adverse effects when introduced in a context where systems constraints are substantial. Finally, the above points suggest that studies of P4P impact on beneficiaries should not only measure utilization of services, but include both health outcomes and a qualitative element, as paying for increased utilization does not necessarily improve health outcomes.

### Study limitations

Our study has a number of limitations. Firstly, only three of the 26 health workers we interviewed for this study were interviewed both before and after the bonus payments were made contrary to the original design. This was due in part to the fact that some of the health workers interviewed in 2010 had moved or were absent during our visit in 2011. Another reason was that we wanted better geographical representation, and therefore decided to cover a larger area for our 2011 interviews in order to capture local variations within the district (i.e. the type of sanctions that had been introduced against home birth). Lastly, study informants were purposively selected and may not be representative of other individuals or settings, hence the study lacks external validity.

## Conclusion

Many scholars have argued that P4P may have adverse effects. This study has contributed to this body of literature by showing how health workers in a low-income setting use coercive strategies in order to reach set targets for deliveries. While P4P in Mvomero may have contributed to an increase in the number of institutional deliveries, the overall health outcomes may not have been positive. Our study has also demonstrated that qualitative studies of P4P interventions should preferably include both community and health worker components since information from community members may be essential in order to ask health workers the right questions – and vice versa. The study has also demonstrated that the P4P programme deviated substantially from the original design when implemented by local authorities – partly due to limited resources, and partly due to fairness ideals that differ from the basic principles of P4P. This lesson is most likely relevant for the nationwide roll-out of P4P programmes in other African countries.

## Competing interests

The authors declare that they have no competing interests. While most of this work was conducted at the Chr. Michelsen Institute and at the Department of Health Promotion and Development (HEMIL), IL is currently employed at Norad’s evaluation department. The evaluation department is an independent body within Norad.

## Authors’ contributions

VC planned and designed the 2010 study as part of his MA thesis in Gender and Development at the University of Bergen [36]. He conducted the data collection in 2010 with the help of a research assistant. VC, IL and SL designed the 2011 study. SL conducted the 2011 interviews with the assistance of two research assistants as part of her post-doc within the project *Strengthening human resources for health: A study of health worker availability and performance in Tanzania* (funded by the Norwegian Research Council). VC had the main responsibility for the data analysis, with assistance from SL. VC and SL developed the first draft of the article. During the review process, IL rewrote the discussion after which IL and SL revised the article critically. All the authors read and approved the final manuscript.

## Pre-publication history

The pre-publication history for this paper can be accessed here:

http://www.biomedcentral.com/1472-6963/14/23/prepub

## References

[B1] Godal T Concept paper for the global business planhttp://www.who.int/pmnch/events/2007/gbpconceptpaper.pdf

[B2] Lauglo M Swai R Payment for performance appraisal: Report to Norad and the Royal Norwegian Embassy, Tanzania 2009 Oslo: Centre for Health and Social Development (HeSo)

[B3] Eichler R Can Pay for performance‚ increase utilization by the poor and improve the quality of health services. Background papers for the Working Group on Performance Based Incentives 2006 Washington DC: Centre for Global Development

[B4] Canavan A Toonen J Elovainio R Performance based financing: an international review of the literature 2008 Royal Tropical Institute (KIT). Development Policy & Practice

[B5] World Bank Results-Based Financing for Health. Brief, Africa Health Forum 2013 Washington DC: World Bank

[B6] Oxman A Fretheim A Can paying for results help to achieve the Millennium Development Goals? Overview of the effectiveness of results-based financing J Evid Based Med 2009 14 2 70 83 10.1111/j.1756-5391.2009.01020.x 21348993

[B7] Meessen B Kashala J-P Musango L Output-based payment to boost staff productivity in public health centres: contracting in Kabutare district, Rwanda Bull World Health Organ 2007 14 108 114 10.2471/BLT.06.032110 17308731 PMC2636284

[B8] Kalk A Paul FA Grabosch E Paying for performance‚in Rwanda: does it pay off? Trop Med Int Health 2010 14 2 182 190 10.1111/j.1365-3156.2009.02430.x 19930141

[B9] Rosenthal MB Frank RG What is the empirical basis for paying for quality in health care? Med Care Res Rev 2006 14 2 135 157 10.1177/1077558705285291 16595409

[B10] Wilson KJ Pay-for-performance in health care: what can we learn from international experience? Ment Health Fam Med 2013 14 1 2 15 0.1097/QMH.0b013e31827dea50 10.1097/QMH.0b013e31827dea50 23271589

[B11] Van Herck P De Smedt D Annemans L Remmen R Rosenthal MB Sermeus W Systematic review: effects, design choices, and context of pay-for-performance in health care BMC Health Serv Res 2010 14 1 247 10.1186/1472-6963-10-247 20731816 PMC2936378

[B12] Basinga P Gertler PJ Binagwaho A Soucat ALB Sturdy J Vermeersch CMJ Effect on maternal and child health services in Rwanda of payment to primary health-care providers for performance: an impact evaluation The Lancet 2011 14 9775 1421 1428 10.1016/S0140-6736(11)60177-3 21515164

[B13] De Walque D Gertler PJ Bautista-Arredondo S Kwan A Vermeersch C de Dieu Bizimana J Using Provider Performance Incentives to Increase HIV Testing and Counseling Services in Rwanda 2013 UC Berkeley: Center for Effective Global Action http://escholarship.org/uc/item/2r69r9ww 10.1016/j.jhealeco.2014.12.001 25554976

[B14] Gertler P Vermeersch C Using Performance Incentives to Improve Medical Care Productivity and Health Outcomes 2013 Massachusetts: National Bureau of Economic Research

[B15] Peabody JW Shimkhada R Quimbo S Solon O Javier X McCulloch C The impact of performance incentives on child health outcomes: results from a cluster randomized controlled trial in the Philippines Health Policy and Planning 2013 [Epub ahead of print] http://www.ncbi.nlm.nih.gov/pubmed/24134922 10.1093/heapol/czt047 PMC4124243 24134922

[B16] Lee JY Lee S-I Jo M-W Lessons from healthcare providers’ attitudes toward pay-for-performance: what should purchasers consider in designing and implementing a successful program? J Prev Med Public Health 2012 14 3 137 147 10.3961/jpmph.2012.45.3.137 22712040 PMC3374963

[B17] Eldridge C Palmer N Performance-based payment: some reflections on the discourse, evidence and unanswered questions Health Policy Plan 2009 14 3 160 166 10.1093/heapol/czp002 19202163

[B18] Eisenhardt KM Agency theory - an assessment and review Acad Manage Rev 1989 14 1 57 74

[B19] Holmstrom B Milgrom P Multitask principal-agent analyses: incentive contracts, asset ownership, and job design J Law Econ Organ 1991 14 24 52 10.1093/jleo/7.special_issue.24

[B20] Frey BS Jegen R Motivation crowding theory: A survey of empirical evidence 2000 University of Zurich: Institute for Empirical Research in Economics

[B21] Das J Hammer JS Leonard K The quality of medical advice in low-income countries J Econ Perspect 2008 14 2 93 114 10.1257/jep.22.2.93 19768841

[B22] Mæstad O Torsvik G Aakvik A Overworked? On the relationship between workload and health worker performance J Health Econ 2010 14 5 686 698 10.1016/j.jhealeco.2010.05.006 20633940

[B23] Lange S Mwisongo A Mæstad O Why don’t clinicians adhere more consistently to Integrated Management of Childhood Illness (IMCI) guidelines? Soc Sci Medin press 10.1016/j.socscimed.2013.12.020 24581062

[B24] Human resources for health in Tanzania: challenges, policy options and knowledge gaps: Chr. Michelsen Institute Report R 2006 Bergen

[B25] Chandler C Chonya S Mtei F Reyburn H Whitty C Motivation, money and respect: a mixed-method study of Tanzanian non-physician clinicians Soc Sci Med 2009 14 11 2078 2088 10.1016/j.socscimed.2009.03.007 19328607

[B26] Songstad NG Rekdal OB Massay DA Blystad A Perceived unfairness in working conditions: the case of public health services in Tanzania BMC Health Serv Res 2011 14 1 34 10.1186/1472-6963-11-34 21314985 PMC3045290

[B27] Eichler R Morgan L Pay for performance in Tanzania 2009 Bethesda, Maryland: Health Systems 20/20 project, Abt. Associates

[B28] The United Republic of Tanzania Payment for performance strategy 2008–2015, Agenda No 5.1 2008 Dar es Salaam: Ministry of Health and Social Welfare

[B29] Songstad NG Lindkvist I Moland KM Chimhutu V Blystad A Assessing performance enhancing tools: experiences with the open performance review and appraisal system (OPRAS) and expectations towards payment for performance (P4P) in the public health sector in Tanzania Global Health 2012 14 1 1 13 10.1186/1744-8603-8-1 22963317 PMC3477072

[B30] The United Republic of Tanzania The Pwani region pay-for-performance (P4P) pilot: design document 2012 Dar es Salaam: Ministry of Health and Social Welfare

[B31] Smithson P Eichler R Winani S Msuya A Olsen T Musch E Results-based bonus design, implementation & budget: ”Malipo kwa Ufanisi Bora katika Huduma za Afya” (MUBHA) 2008 Dar es Salaam: Ministry of Health and Social Welfare of Tanzania

[B32] National Bureau of Statistics (NBS), ICF Macro Demographic and Health Survey 2010 2011 Dar es Salaam: NBS and ICF Macro

[B33] The United Republic of Tanzania Mvomero District Council: Comprehensive Council Health Plan July 2009 - June 2010 2009 Dar es Salaam: Prime Minister’s Office- Regional Administration and Local Government

[B34] Kvale S Doing interviews 2007 London: SAGE

[B35] The United Republic of Tanzania Human resource for health strategic plan 2008–2013 2008 Dar es Salaam: Ministry of Health and Social Welfare

[B36] Chimhutu V Pay for performance in maternal health in Tanzania: perceptions, expectations and experiences in Mvomero district 2011 Masters thesis. University of Bergen: Department of Health Promotion and Development

[B37] Mrisho M Schellenberg AJ Mushi KA Obrist B Mshinda H Tanner M Factors affecting home delivery in rural Tanzania Trop Med Int Health 2008 14 862 872 10.1111/j.1365-3156.2007.01855.x 17596254

[B38] Kahabuka C Kvale G Moland KM Hinderaker SG Why caretakers bypass Primary Health Care facilities for child care - a case from rural Tanzania BMC Health Serv Res 2011 14 315 10.1186/1472-6963-11-315 22094076 PMC3234197

[B39] Hartmann B The return of population control: incentives, targets and the backlash against Cairo DifferenTakes Spring 2011 14 1 4

[B40] Magrath P Nichter M Paying for performance and the social relations of health care provision: an anthropological perspective Soc Sci Med 2012 14 2012 10.1016/j.socscimed.2012.07.025 22921245

[B41] Ifakara Health Institute P4P Process Round One Report: An Assessment of Activities Implemented from the Introduction of the Scheme in January 2011- up to March 2012 2012 Dar es Salaam: Ifakara Health Institute

[B42] Cappelen AW Sorensen EO Tungodden B Responsibility for what? Fairness and individual responsibility Eur Econ Rev 2010 14 3 429 441 10.1016/j.euroecorev.2009.08.005

[B43] Nozick R Anarchy, State and Utopia 1974 New York: Basic books

